# Noise-induced stabilization and fixation in fluctuating environment

**DOI:** 10.1038/s41598-018-27982-1

**Published:** 2018-06-27

**Authors:** Immanuel Meyer, Nadav M. Shnerb

**Affiliations:** Department of Physics, Bar-Ilan University, Ramat-Gan, IL52900 Israel

## Abstract

The dynamics of a two-species community of *N* competing individuals are considered, with an emphasis on the role of environmental variations that affect coherently the fitness of entire populations. The chance of fixation of a mutant (or invading) population is calculated as a function of its mean relative fitness, the amplitude of fitness variations and their typical duration. We emphasize the distinction between the case of pairwise competition and the case of global competition; in the latter a noise-induced stabilization mechanism yields a higher chance of fixation for a single mutant. This distinction becomes dramatic in the weak selection regime, where the chance of fixation for a single deleterious mutant is an *N*-independent constant for global competition and decays like (ln *N*)^−1^ in the pairwise competition case. A Wentzel-Kramers-Brillouin (WKB) technique yields a general formula for the chance of fixation of a deleterious mutant in the strong selection regime. The possibility of long-term persistence of large [${\mathscr{O}}$(*N*)] suboptimal (and extinction-prone) populations is discussed, as well as its relevance to stochastic tunneling between fitness peaks.

## Introduction

A fundamental problem in the fields of population genetics, evolution, and community ecology, is to predict the fate of a single mutant (or invader) introduced in a finite population of wild types. For a fixed-size community of *N* individuals, with Markovian, zero-sum dynamic driven by stochastic birth-death events, the mutant population eventually reaches either fixation or extinction. The classical analysis, provided by Kimura and his successors^1,2^, is focused on the neutral case, (where the dynamic is only due to demographic stochasticity, i.e., the noise inherent to the birth-death process), and on *time*-*independent* selective forces (deleterious/beneficial mutation).

When the system is neutral (no fitness differences, all individuals are demographically equivalent) the chance of a single mutant to reach fixation is, by symmetry, Π_*n*=1_ = 1/*N*. In general when the mutant population has abundance *n*, Π_*n*_ = *n*/*N*.

Under fixed selection *s,* the fixation probability is,

1 $${{\rm{\Pi }}}_{n}=\frac{1-{e}^{-sn}}{1-{e}^{-Ns}}.$$

In the weak selection regime, $N|s|\ll 1$ and $|s|\ll 1$, the effect of selection is negligible and the neutral result reemerges. When the mutation is beneficial (*s* > 0, but still $s\ll 1$) and $Ns\gg 1$ (strong selection regime), Π_*n*=1_ is *N*-independent and converges, at large *N*, to *s*. A simple and intuitive argument for this result relies on the distinction between the region 1 ≤ *n* ≤ 1/*s*, where demographic fluctuations are dominant and the dynamic is more or less neutral, and the region 1/*s* < *n*, where selection dominates and fixation is almost assured. The chance of fixation is thus determined by the chance to cross a neutral region of length 1/*s*, which is exactly *s*^3^.

When the mutation is deleterious (*s* < 0), Π_*n*=1_ decays exponentially with *N*|*s*| in the strong selection regime, since now fixation is reached through a series of unlikely events against a constant bias. Accordingly, for any practical purpose one may neglect the chance of a deleterious mutation to reach fixation when *N* is large. This observation poses a serious question to the standard theory of species evolution. If genotypes of existing species are associated with local maxima in the fitness landscape, evolutionary pathways must cross fitness valleys. Because the chance of such “tunneling” events is vanishingly small, the timescales associated with it turn out to be unrealistically high^4^.

This set of results was obtained for a system with pure demographic noise, where the stochastic component in the reproductive success of each individual is independent of the success of its conspecific. As a result, the per-generation noise-induced abundance variations scale like the square root of the population size [i.e., are ${\mathscr{O}}(\sqrt{n})$]. Environmental changes that affect coherently the fitness of entire populations lead to much stronger, ${\mathscr{O}}(n)$, abundance variations^5^, therefore one would expect a substantial impact of these fluctuations on the chance of fixation. Recently, many empirical studies showed that the effect of coherent fitness fluctuations is indeed much more pronounced than that of the demographic noise^6–9^, and that selection changes its amplitude and sign through time^10,11^. Consequently, the study of temporal environmental stochasticity received considerable attention^12–21^. In parallel, a few recent experimental studies have considered the response of microorganism communities (and their evolutionary pathways) to fitness fluctuations^22–24^.

In some scenarios fluctuating selection may activate a noise-induced stabilizing mechanism^25^. The main aim of this work is to calculate fixation probabilities in that case and to contrast them with the known results that were obtained in the absence of such a stabilizing mechanism. As we shall see, the fixation probabilities change dramatically under the influence of this mechanism. Moreover, in that case the system appears to allow for long-term persistence of suboptimal mutant populations, a phenomenon that may facilitate stochastic tunneling through fitness valleys (see discussion section).

In the next section we discuss the two models considered throughout this paper and clarify the conditions under which noise-induced stabilization occurs. In the third section we define mathematically these two models and explain the methodology used to obtain the chance of fixation when the environment fluctuates. The results are presented in the forth and the fifth sections and the possibility of stochastic tunneling is considered in the discussion. A glossary is provided in Table 1 and the main results of this paper are summarized in Table 2.

**Table 1:** Glossary.

Term	Description
*N*	number of individuals in the community (both species).
*n*	number of individuals belonging to the mutant population.
*x*	fraction of mutants, *x* = *n*/*N* (1 − *x* is the fraction of wild type).
*s*_0_	the time-independent component of the fitness.
*γ*	the amplitude of fitness fluctuations.
*δ*	correlation time of the environment, measured in generations.
$g\equiv \delta {\gamma }^{2}/2$	the strength of environmental fluctuations.
$G\equiv N\delta {\gamma }^{2}/2=Ng$	environmental stochasticity *g* in units of demographic noise 1/*N*.
$\tilde{s}\equiv 2{\eta }_{0}/{\gamma }^{2}$	scaled selection.
$\alpha \equiv {s}_{0}/g=\tilde{s}/\delta$	useful derived parameter.

**Table 2:** A summary of the main results obtained in this paper.

	Pure demographic *γ* = 0	Model A	Model B
Π(*x*)	Eq. (1)	Eqs (11) and (12)	Eq. (23)
*n*_*c*_	1/s	$\frac{\exp (g/\,2\,|\,{s}_{0}|)}{g}$	$\frac{\exp (g/\,2\,|\,{s}_{0}|)}{g}$
Π_*n*=1_	$\frac{1-{e}^{-s}}{1-{e}^{-Ns}}$	$\frac{1-{\mathrm{(1}+g)}^{-{s}_{0}/g}}{1-{G}^{-2\alpha }}$	$\frac{1-{\mathrm{(1}+g)}^{-\frac{1}{\delta }(\tilde{s}+\mathrm{1)}}}{1+{D}_{1}{G}^{-2\alpha }}$
Π_*n*=1_ Weak selection	$\frac{1}{N}$	$\frac{\mathrm{ln}\,\mathrm{(1}+g)}{2\,\mathrm{ln}\,Ng}$	$\frac{1-{\mathrm{(1}+g)}^{-\frac{1}{\delta }(\tilde{s}+\mathrm{1)}}}{2}$
${{\rm{\Pi }}}_{n=1}^{{s}_{0} > 0}$ Strong selection	*s*	$1-\frac{1}{{(1+g)}^{{s}_{0}/g}}$	$1-\frac{1}{{(1+g)}^{\frac{1}{\delta }(\tilde{s}+1)}}$
${{\rm{\Pi }}}_{n=1}^{{s}_{0} < 0}$ Strong selection	*e* ^−*N*|*s*|^	*N* ^−2*f* ′^	*N* ^−2*f* ′^

In the last line, *f* ′ is the solution of the transcendental Equation (30), and a few approximations for it are given in the sixth section.

## Noise-Induced Stabilization

To begin, let us define two zero-sum competition models, one that does not allow for noise-induced stabilization when the environment fluctuates (model A) and one that admits this phenomenon (model B).

In *model A* competition is pairwise and selection acts linearly. As an example one may envisage a population of competing animals, where a random encounter between two of them may end up in a struggle over, say, a piece of food, a mate or a territory. To model such a system we assume that these “duels” occur at a constant rate between two randomly picked individuals and in each duel the loser dies and the winner produces a single offspring. If *x* = *n*/*N* is the population fraction of the mutant, the chance of an interspecific “duel” is 2*x*(1 − *x*) and the chance of the mutant individual winning such a duel is defined to be 1/2 + *s*/4. Accordingly, the deterministic growth/decay of *x* (when time is measured in generations, *N* elementary duels in each generation) satisfies the logistic equation,

2 $$\dot{x}=2x(1-x)([\frac{1}{2}+\frac{s}{4}]-[\frac{1}{2}-\frac{s}{4}])=sx(1-x).$$

If *s* fluctuates in time such that its mean value is zero (a time-averaged neutral model^16^) the system performs an unbiased random walk along the *z* = ln[*x*/(1 − *x*)] axis. When the mean of *s*, *s*_0_, is nonzero, the random walk is biased towards either fixation or extinction.

In *model B*, on the other hand, the competition is global. In a forest, for example, following the death of an adult tree local seeds or seedlings are competing for the opened gap. If the seed dispersal length is larger than the linear size of the forest, the seed bank at any given location reflects the composition of the whole community. Death events are assumed to be random and fitness-independent. Accordingly, the chance of a mutant species with relative log-fitness *s* to gain one individual in an elementary death-birth event is a multiplication of the chance that a wild-type was chosen to die, 1 − *x*, by the chance of the mutant to win the gap, *xe*^*s*^/(1 − *x* + *xe*^*s*^) (a Moran process). The probability of the mutant species to lose one individual is, accordingly, (1 − *x*)*x*/(1 − *x* + *xe*^*s*^). In contrast with model A, here the fitness dependence is nonlinear. As a result, the deterministic dynamic satisfies,

3 $$\dot{x}=\frac{x(1-x)\,({e}^{s}-1)}{1-x+x{e}^{s}}\approx sx(1-x)+{s}^{2}x(1-x)\,(1/2-x),$$

where the last term comes from a second order expansion in *s*.

While the linear (*s*) term in Eq. (3) gives, as in Eq. (2), a flow towards zero or one, the *s*^2^ term has an attractive fixed point at *x* = 1/2. When *s* is fixed in time this second term is negligible, but when *s* fluctuates the *s*^2^ term may dominate. Therefore, in model B environmental variations may induce stability through nonlinear fitness dependence^25^.

While for pairwise competition (model A) the effect of environmental fluctuations weakened when their correlation time decreases, the stabilizing effect of the global competition model reaches its maximum when the correlation time is minimal^18^.

The ability of environmental fluctuations to stabilize a coexistence state was pointed out by Chesson and coworkers^25,26^ and is known in the ecological literature as the storage effect. The storage effect stabilizes a coexistence state when the fitness affects recruitment but death occurs at random. This is the situation in our model B, which is an individual based version of the lottery game considered by Chesson and Warner^25^, see a detailed discussion in^17^. On the other hand, in model A fitness affects both birth and death in an anticorrelated manner. As a result there is no storage effect stabilization in that case. Models A and B are thus the two extreme scenarios; in general, as long as the effect of fitness on recruitment is larger than its effect on death, one should expect the stabilizing mechanism to affect the system.

## Model Definitions and the Backward Kolmogorov Equation

For both model A and model B we assume that

$$s(t)={s}_{0}+\zeta (t),$$

where *s*_0_ is the mean log-fitness and *ζ*(*t*) may take two values, ±*γ* (telegraphic, or dichotomous, noise). Both white Gaussian noise and white Poisson noise can be recovered from the dichotomous noise by taking suitable limits^27^, so the results obtained here are quite generic.

The chance of the environment to switch (from ±*γ* to $\mp$*γ*) is 1/(*δN*) per elementary birth-death event, so the sojourn time of the environment is taken from a geometric distribution with mean *δN* elementary birth-death steps, or *δ* generations. The chance of fixation when the mutant is in the plus (minus) state and its abundance is *n*, Π_+,*n*_ (Π_−,*n*_), satisfies the discrete backward Kolmogorov equation (BKE),

4 $$\begin{array}{rcl}{{\rm{\Pi }}}_{+,n} & = & (1-\frac{1}{N\delta })\,[{W}_{+,n}{{\rm{\Pi }}}_{+,n+1}+{W}_{-,n}{{\rm{\Pi }}}_{+,n-1}+(1-{W}_{+,n}-{W}_{-,n})\,{{\rm{\Pi }}}_{+,n}]\\  &  & +\,\frac{1}{N\delta }[{Q}_{+,n}{{\rm{\Pi }}}_{-,n+1}+{Q}_{-,n}{{\rm{\Pi }}}_{-,n-1}+(1-{Q}_{+,n}-{Q}_{-,n})\,{{\rm{\Pi }}}_{-,n}]\\ {{\rm{\Pi }}}_{-,n} & = & (1-\frac{1}{N\delta })\,[{Q}_{+,n}{{\rm{\Pi }}}_{-,n+1}+{Q}_{-,n}{{\rm{\Pi }}}_{-,n-1}+(1-{Q}_{+,n}-{Q}_{-,n})\,{{\rm{\Pi }}}_{-,n}]\\  &  & +\,\frac{1}{N\delta }[{W}_{+,n}{{\rm{\Pi }}}_{+,n+1}+{W}_{-,n}{{\rm{\Pi }}}_{+,n-1}+(1-{W}_{+,n}-{W}_{-,n})\,{{\rm{\Pi }}}_{+,n}]\end{array}$$

where *W*_±_s are the transition probabilities in the +*γ* state and the *Q*_±_s are the corresponding probabilities in the (−*γ*) states. The transition probabilities of model A are (we replaced *n*/*N* by *x*),

5 $$\begin{array}{ll}{W}_{+,n}=x(1-x)\frac{2+{s}_{0}+\gamma }{2} & {W}_{-,n}=x(1-x)\frac{2-{s}_{0}-\gamma }{2}\\ {Q}_{+,n}=x(1-x)\frac{2+{s}_{0}-\gamma }{2} & {Q}_{-,n}=x(1-x)\frac{2-{s}_{0}+\gamma }{2},\end{array}$$

and the corresponding probabilities for model B are,

6 $$\begin{array}{ll}{W}_{+,n}=\frac{(1-x)x{e}^{\gamma +{s}_{0}}}{x{e}^{\gamma +{s}_{0}}+(1-x)} & {W}_{-,n}=\frac{x(1-x)}{x{e}^{\gamma +{s}_{0}}+(1-x)}\\ {Q}_{+,n}=\frac{(1-x)x{e}^{{s}_{0}}}{x{e}^{{s}_{0}}+(1-x){e}^{\gamma }} & {Q}_{-,n}=\frac{x(1-x){e}^{\gamma }}{x{e}^{{s}_{0}}+(1-x){e}^{\gamma }}.\end{array}$$

The exact difference equation (4), with the appropriate set of *W*’s and *Q*’s and with the boundary conditions Π_+,0_ = Π_−,0_ = 0, Π_+,*N*_ = Π_−,*N*_ = 1, may be solved numerically as a Markov chain^28^ or as a linear system^18^, and these solutions are compared below with the analytic formulas.

One can translate this pair of discrete BKEs for Π_±,*n*_ into an equivalent set for Π_*n*_ ≡ (Π_+,*n*_ + Π_−,*n*_)/2 and Δ_*n*_ ≡ (Π_+,*n*_ − Π_−,*n*_)/2. Taking the continuum limit where *n* is replaced by *Nx* and functions of *x* ± 1/*N* are expanded to second order in 1/*N*, a pair of coupled, second order differential equations for Π(*x*) and Δ(*x*) emerges. In^18^ we have analyzed these equations in the limit of large *N* and small *s*_0_. Using a dominant balance argument we showed that the dynamic is governed by a single second-order equation (in^18^ only the time to absorption was discussed, but the equation for Π is the homogenous version of the corresponding BKE, see^29^). This equation is,

7 $$[1+Gx(1-x)]{\rm{\Pi }}^{\prime\prime} (x)+({s}_{0}N+\eta G(1-2x))\,{\rm{\Pi }}^{\prime} (x)=0,$$

with the boundary conditions Π(0) = 0 and Π(1) = 1. Here $g\equiv {\gamma }^{2}\delta /2$ is the strength of the environmental noise and $G\equiv Ng$ is this strength divided by the strength of demographic stochasticity, 1/*N*. The differences between model A and B are encapsulated in the parameter *η*:

8 $$\begin{array}{ll}{\rm{Model}}\,{\rm{A}} & \eta =1\\ {\rm{Model}}\,{\rm{B}} & \eta =1+\frac{1}{\delta }.\end{array}$$

As *δ* grows, model B becomes closer to model A. However, the derivation of Eq. (7) assumes that fixation cannot occur during a single sweep of the environment, so an increase in *δ* is legal only if *N* increases such that $\delta \ll \,\mathrm{ln}\,N/({s}_{0}+\gamma )$.

Eq. (7) may be solved using integrating factors, but this leads to complicated and hard to interpret nested integral expressions. Instead one may analyze this equation in the inner ($x\ll 1$), middle ($Gx\mathrm{(1}-x)\gg 1$) and outer ($1-x\ll 1$) regimes and then match asymptotically the solutions in the large *N* (more precisely, large *G*) limit. In the next section we present briefly the results for model A, following^30^. Our purpose is to contrast these result with the outcomes of model B and to emphasize the effects of the noise-induced stabilizing mechanism.

## Model A: Local Competition and Linear Selection

The solutions of model A in the inner, middle and outer regimes are given by,

9 $$\begin{array}{rcl}{{\rm{\Pi }}}_{in}(x) & = & {C}_{1}(1-{(1+Gx)}^{-\alpha })\\ {{\rm{\Pi }}}_{m}(x) & = & {C}_{3}+{C}_{2}{(\frac{1-x}{x})}^{\alpha }\\ {{\rm{\Pi }}}_{out}(x) & = & 1-{C}_{4}(1-{[1+G(1-x)]}^{\alpha }),\end{array}$$

where $\alpha \equiv {s}_{0}/g$ and the constants, that were determined using asymptotic matching, are,

10 $$\begin{array}{rcl}{C}_{1} & = & {C}_{3}=\frac{1}{(1-{G}^{-2\alpha })}\\ {C}_{4} & = & 1-{C}_{1}\\ {C}_{2} & = & -\,{C}_{1}{G}^{-\alpha }.\end{array}$$

For |*α*| < 1 one may use the uniform approximation solution for an arbitrary *x*,

11 $${{\rm{\Pi }}}_{unif}(x)={C}_{1}\,(1-{(1+Gx)}^{-\alpha }-{[1+G(1-x)]}^{\alpha }+\tfrac{1-({x}^{\alpha }-1)\,{(1-x)}^{\alpha }}{{(Gx)}^{\alpha }})+{[1+G(1-x)]}^{\alpha }.$$

For |*α*| > 1, if *C*_2_ is negligible, i.e., if ${G}^{-\alpha }\ll 1$, the uniform approximation takes the form,

12 $${{\rm{\Pi }}}_{unif}(x)={C}_{1}\,(1-{(1+Gx)}^{-\alpha }-{[1+G(1-x)]}^{\alpha })+{[1+G(1-x)]}^{\alpha }.$$

**Figure 1:**
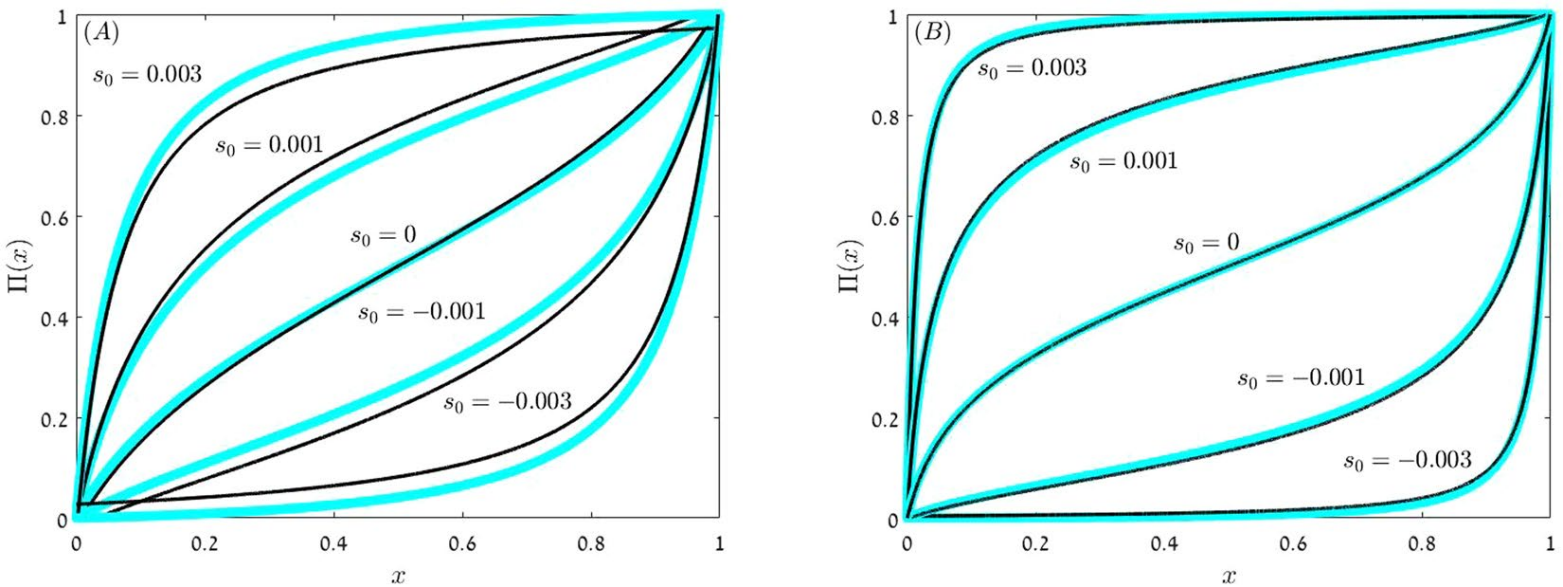
Π(*x*) vs. *x* for model A. In both panels *γ* = 0.2 and *δ* = 0.1; *N* = 5,000 in panel (A) and *N* = 20,000 in panel (B). Numerical solutions of the discrete equation (4) (blue circles) are compared with the uniform approximations (11) and (12) (black full lines) for *s*_0_ = 0 (*α* = 0), *s*_0_ = ±0.001 (*α* = 1/2) and *s*_0_ = ±0.003 (*α* = 3). When |*α*| < 1 (11) has been used, while for |*α*| > 1 we implemented the uniform approximation (12). For any fixed nonzero value of *s*_0_, as *N* grows Π(*x*) sticks to either one (if *s*_0_ > 0) or zero (if *s*_0_ < 0) in the middle and the outer regions. The accuracy of the uniform approximation becomes better when *N* increases.

The agreement between Π_*unif*_ and the outcomes of the numerical solutions of Eq. (4) is demonstrated in Fig. 1. The theory and the numerics become closer and closer as *N* increases.

The chance of a single mutant to reach fixation is obtained by plugging *x* = 1/*N* into the inner solution,

13 $${{\rm{\Pi }}}_{n=1}=\frac{1-\frac{1}{{(1+g)}^{{s}_{0}/g}}}{1-{G}^{-2\alpha }}.$$

**Figure 2:**
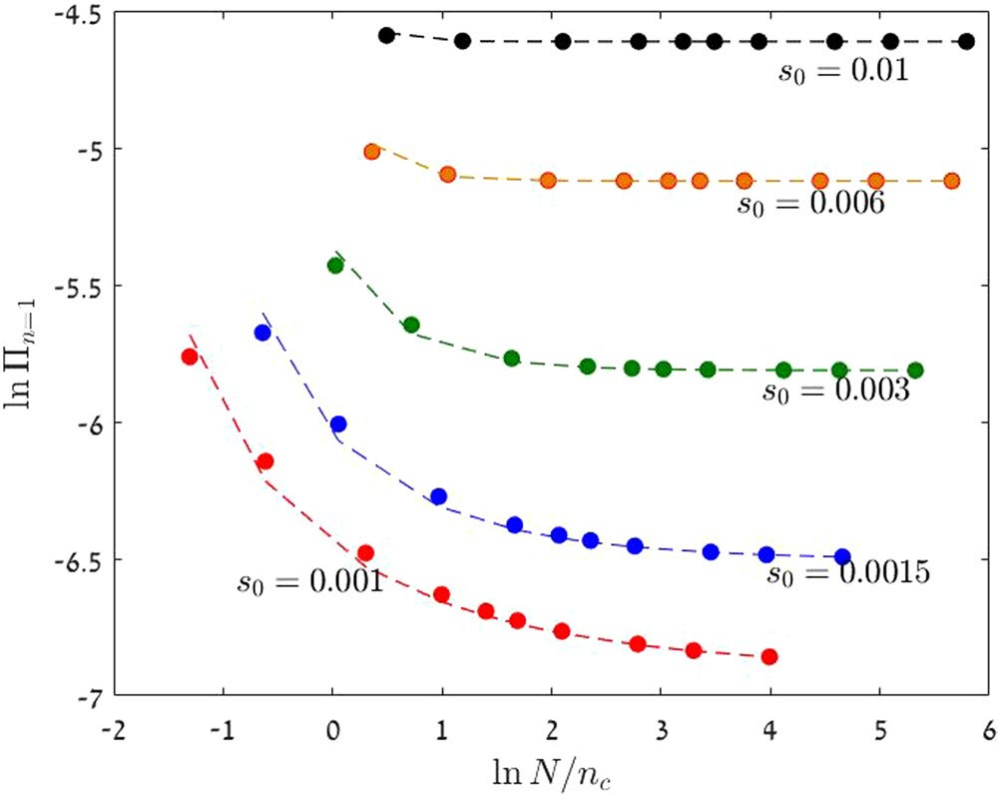
The chance of fixation by the lineage of a single beneficial mutant, Π_*n*=1_, is plotted against the effective community size *N*/*n*_*c*_ on a double logarithmic scale. Parameters are *γ* = 0.2, *δ* = 0.2 and different values of *s*_0_. Filled circles represent numerical solutions and the dashed lines are the prediction of Eq. (13). The actual values of *N* used in this figure span four orders of magnitude, from 10 to 10^5^. For *N* < *n*_*c*_ the chance of fixation decays logarithmically with *N* and Π_*n*=1_ saturates to a finite value when *N* > *n*_*c*_.

In Fig. 2 the predictions of (13) are shown to fit the numerical results.

The most important conclusion from the comparison between Eqs (9 and 10) and (1) is the modification of the criteria for strong selection. We *define* the strong selection sector as the parameter regime where the chance of fixation of a single beneficial mutant becomes *N* independent. This happens when $N\gg {n}_{c}$ where *n*_*c*_ marked the point where the deterministic effect of selection dominates against the stochastic effects of fluctuations. While for system with selection and pure demographic noise *n*_*c*_ = 1/*s*, here the criteria for strong selection is *C*_1_ = *C*_3_ = 1, i.e.,

14 $${n}_{c}\sim \frac{\exp (g/2|{s}_{0}|)}{g}.$$

This scale diverges exponentially when the mean selection is much smaller than the effective strength of fitness fluctuations, which might be the generic situation in living systems. Accordingly, under environmental stochasticity systems may be in the weak selection regime even if *N* is very large.

Two other characteristic scales in this system are *N*_1_ and *n*_2_. For $N\gg {N}_{1}$, *C*_2_ = 0, so ${N}_{1} \sim \exp (g/{s}_{0})/g$. The chance of fixation of the mutant population becomes large when it reaches *n*_2_ such that Π_*in*_(*n*_2_/*N*) > 1 − *e*^−1^, thus,

15 $${n}_{2}\equiv \frac{{\exp }^{g/{s}_{0}}-1}{g}.$$

This scale has been identified in^15^. Clearly, all three scales have similar features as they grow exponentially when *s*_0_ is much smaller than *g*. For a system with pure demographic noise and selection (Eq. 1), the chance of fixation becomes *N* independent above *n*_*c*_ = 1/*s*_0_ and the condition for *n*_2_ yields 1/*s*_0_ as well. While *n*_2_ of (15) converges to this limit when *g* → 0, *n*_*c*_ does not, and this reflect the inadequacy of our expression for *C*_1_ in the ${s}_{0}\gg \gamma$ limit^30^.

In the weak selection regime, *N* < *n*_*c*_, the chance of fixation is *N*-dependent. When $\alpha \,\mathrm{ln}\,G\ll 1$ one can expand the inner solution in small *α* to obtain^15,30^,

16 $${{\rm{\Pi }}}_{n=1}=\frac{\mathrm{ln}(1+g)}{2\,\mathrm{ln}\,G}.$$

While $\alpha \,\mathrm{ln}\,G\ll 1$ is small, ln *G* may be large, so in the weak selection regime, and in particular in the time-averaged neutral scenario where *s*_0_ = 0, the chance of fixation decays logarithmically with system’s size as demonstrated in Fig. 2.

When selection is weak an increase in *g* increases the chance of fixation. In the purely demographic neutral case Π is determined by abundance so Π_*n*=1_ = 1/*N*. In the limit of infinitely strong environmental variations a mutant will reach fixation for certainty if it was born in the right time, so the chance of fixation will grow to one half. In general the transition from abundance-dependence to environment dependence facilitates the chance of low-abundance populations to win^15^.

The situation is completely different in the strong selection regime^30^, where Π_*n*=1_ is a monotonously *decreasing* function of *g*. Here the reason is the divergence of *n*_*c*_ when *g* increases, meaning that the chance of the beneficial mutant population to enter the region of deterministic selective growth is much smaller.

## Model B: Noise Induced Stabilization

In model B the dynamic is affected by the noise-induced stabilizing mechanism that facilitates the establishment of a mutant. Before we introduce the expressions for the chance of fixation, we would like to discuss the conditions under which this stabilizing mechanism takes place.

When *s*(*t*) = *s*_0_ ± *γ*, as described above, and the environmental fluctuations are rapid, Eq. (3) for the deterministic dynamic of the population takes the form

17 $$\dot{x}\approx {s}_{0}x(1-x)+({s}_{0}^{2}+{\gamma }^{2})x(1-x)\,(1/2-x).$$

Assuming, $|{s}_{0}|\ll \gamma$, this equation supports an attractive fixed point at

18 $${x}^{\ast }=1/2+{s}_{0}/{\gamma }^{2},$$

and x^*^ is between zero and one if

19 $$-\,1 < \tilde{s}\equiv \frac{2{s}_{0}}{{\gamma }^{2}} < 1.$$

Therefore, $\tilde{s}$, the ratio between mean selection and environmental fluctuations, determines the qualitative behavior of the system. When $|\tilde{s}| < 1$ the noise induces a stable coexistence point and the dynamic of model B differs substantially from the dynamic of model A. When $|\tilde{s}| > 1$ the deterministic force does not change its sign in the region between fixation and extinction, so the behaviors of model A and model B are qualitatively similar. In agreement with this observation, in^18^ the time to absorption (either fixation or extinction) for model B was found to diverge like ${N}^{\mathrm{(1}-|\tilde{s}|)/\delta }$ when *N* is large and $|\tilde{s}| < 1$, while for $|\tilde{s}| > 1$ the *N* scaling is sublinear. Therefore, in this section we consider only the $|\tilde{s}| < 1$ case.

Implementing the technique of asymptotic matching to model B when $|\tilde{s}| < 1$, the solutions for Eq. (7) with *η* = 1 + 1/*δ* are,

20 $$\begin{array}{rcl}{{\rm{\Pi }}}_{in}(x) & = & {C}_{1}[1-{(1+Gx)}^{-\alpha -1/\delta }]\\ {{\rm{\Pi }}}_{m}(x) & = & {C}_{3}+{C}_{2}\,{\int }^{x}\frac{{(1-t)}^{\alpha -\eta }}{{t}^{\alpha +\eta }}dt\\ {{\rm{\Pi }}}_{out}(x) & = & 1-{C}_{4}(1-{[1+G(1-x)]}^{\alpha -1/\delta },\end{array}$$

where as before $\alpha \equiv {s}_{0}/g$ and the constants are given by,

21 $$\begin{array}{rcl}{C}_{1} & = & {C}_{3}=\frac{1}{(1+{D}_{1}{G}^{-2\alpha })}\\ {C}_{4} & = & 1-{C}_{1}\\ {C}_{2} & = & (\alpha +1/\delta ){C}_{1}{G}^{-\alpha -1/\delta },\end{array}$$

with

22 $${D}_{1}=\frac{1/\delta +\alpha }{1/\delta -\alpha }=\frac{1+\tilde{s}}{1-\tilde{s}}.$$

**Figure 3:**
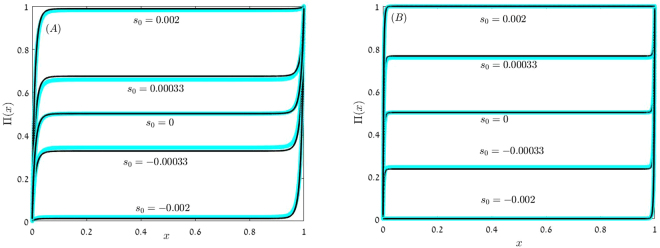
Π(*x*) vs. *x* for model B. In both panels *γ* = 0.2 and *δ* = 0.1, in panel (A) *N* = 5000 and in panel (B) *N* = 20,000. Numerical solutions of the discrete equation (4) (blue circles) are compared with the uniform approximations (23) (full black lines) for *s*_0_ = 0, *s*_0_ = ±0.00033 and *s*_0_ = ±0.002. The pronounced plateau in which Π(*x*) = *C*_1_, where *C*_1_ is neither zero nor one, exists when $|\tilde{s}| < 1$. As *N* growth the value of *C*_1_ increases (for positive *s*_0_) or decreases (for negative *s*_0_), as one may notice by comparing the lines for *s*_0_ = ±0.00033 in the two panels.

The main difference between Eqs (20) and (21) and their model A counterparts, Eqs (9) and (10), is the different scaling of *C*_2_. In model B, *C*_2_ goes to zero when $N\gg \exp (\delta )/g$. Above this *s*_0_-independent point, the chance of fixation in the middle region is fixed, *C*_3_ = *C*_1_, as demonstrated in Fig. 3. A wide plateau emerges in the middle region due to the force towards x^*^. This force is strong, hence Π becomes almost *x*-independent.

The uniform solution when *C*_2_ → 0 has a relatively simple form (see Fig. 3),

23 $${\rm{\Pi }}(x)={C}_{1}[1-{(1+Gx)}^{-\alpha -1/\delta }-{[1+G(1-x)]}^{\alpha -1/\delta }]+{[1+G(1-x)]}^{\alpha -1/\delta },$$

and the chance of fixation of a single mutant (*x* = 1/*N*) is,

24 $${{\rm{\Pi }}}_{n=1}=\frac{1-\frac{1}{{(1+g)}^{\frac{1}{\delta }(\tilde{s}+1)}}}{1+{D}_{1}{G}^{-2\alpha }}.$$

**Figure 4:**
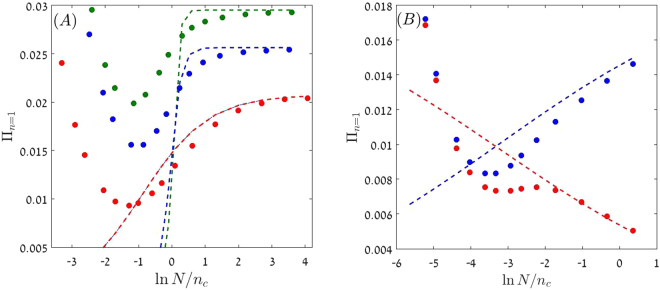
The chance of fixation for the lineage of a single mutant, Π_*n*=1_, is plotted against the effective community size *N*/*n*_*c*_ on a semi-logarithmic scale (the *y* axis is linear, as opposed to Fig. 2). Parameters are *γ* = 0.2 and *δ* = 0.1, so *G* = *Ng* = 1 corresponds to *N* = 500, which is the seventh point in each dataset. Filled circles represent the results of a numerical simulation and the dashed lines are the prediction of Eq. (24). The actual values of *N* used here are between 10 to 20,000 (for *s*_0_ = 0.001, *N* goes up to 80,000). In panel (A) the results are shown for *s*_0_ = 0.001 (red) *s*_0_ = 0.06 (blue) and *s*_0_ = 0.01 (green). The chance of fixation *grows* with *N* and becomes *N* independent in the strong selection regime. Panel (B) shows the results for the weak selection ($N\ll {n}_{c}$) regime for both positive and negative selection, *s*_0_ = 0.0003 (blue) and *s*_0_ = −0.0003 (red). The linear growth/decay of *Pi* with ln *N* reflects the first order correction to Π as calculated in Eq. (26).

Amazingly, Π_*n*=1_ (for a beneficial mutant) turns out to be an *increasing* function of *N*, a behaviour that manifests itself in Fig. 4. This phenomenon reflects the stabilizing effect of the nonlinear mechanism: the chance to reach the plateau does not depend on *N* because the plateau occurs at values of *x* that scale like 1/*N*. For example, in the large *N* limit ${{\rm{\Pi }}}_{{s}_{0}=0}(x)$ sticks to 1/2 for any *x*(1 − *x*) > 2/(*Nγ*^2^). For $G=Ng\ll 1$ the chance of fixation decays like 1/*N* since this regime (in which our expressions fail) is dominated by demographic stochasticity. When *N* increases the stabilizing mechanism wins against the demographic noise and leads to an increase of the chance of fixation.

Π_*n*=1_ in Eq. (24) is a multiplication of two factors: its numerator is the chance of establishment Π_*est*_, which is the probability that the mutant population will reach the basin of attraction of the coexistence fixed point (the plateau). *C*_1_, that determines the denominator, is the chance that the mutant population will reach fixation given establishment.

If the mutant is advantageous (*s*_0_ > 0) and the system is in its strong selection regime (*G*^−2*α*^ → 0 or $N\gg {n}_{c}$), *C*_1_ = 1 and,

25 $${{\rm{\Pi }}}_{n=1}\approx {{\rm{\Pi }}}_{est}=1-\frac{1}{{(1+g)}^{\frac{1}{\delta }(\tilde{s}+1)}}.$$

This is a monotonously decreasing function of *δ*. When *δ* increases, the stochasticity becomes stronger and the stabilizing mechanism weakens^17^, both effects tend to decrease the chance of fixation. The dependence on the amplitude of environmental fluctuations, *γ*, is more complicated. When *γ* is small, its increase facilitates the stabilizing mechanism that increases the chance of fixation, while for large *γ* the increase in *n*_*c*_ is the dominant effect and Π_*n*=1_ decreases.

Model A and model B differ even more dramatically in the weak selection regime $N\ll {n}_{c}$, where *G*^−*α*^ ≈ 1 − *α* ln *G*. For model B, the chance of fixation becomes,

26 $${{\rm{\Pi }}}_{n=1}\approx \frac{1-{(1+g)}^{-\frac{1}{\delta }(\tilde{s}+1)}}{2}(1+\frac{\tilde{s}\,\mathrm{ln}(Ng)}{\delta }).$$

Unlike model A, where the chance of fixation decays logarithmically with *N* in the weak selection regime, for model B in the same case the chance of fixation is ***N***-**independent**. As long as $\tilde{s}\,\mathrm{ln}(Ng)/\delta \ll 1$ the chance of fixation is simply one half of the chance of establishment: the effective strength of the selection bias, $\tilde{s}$, in that case is zero, so once the mutant population reaches the plateau its odds to win or to lose are equal. For nonzero $\tilde{s}$ there is a linear increase or decrease of Π_*n*=1_ as a function of ln *N*. This relatively weak effect is demonstrated in panel (B) of Fig. 4.

Before concluding this section we would like to add a technical comment about our numerical calculations. Π(*x*) is obtained from Eq. (4) by solving a linear problem (dividing a matrix by a vector). Using the sparsity of the matrix, in model A we were able to analyze systems with up to *N* = 10^6^ individuals. Because of the plateau that characterizes model B in the strong selection regime, this numerical solution becomes difficult; the plateau indicates that the matrix to be inverted is almost singular. To overcome this problem we have used quadrapole precision algorithm and this makes the numerics much slower and limits available system sizes to *N* values up to 20,000.

## The Chance of Fixation For A Deleterious Mutant Under Strong Selection - A WKB Approach

Until now we discussed the weak selection regime for both beneficial (*s*_0_ > 0) and deleterious (*s*_0_ < 0) mutants, but the strong selection regime ($N\gg {n}_{c}$) for deleterious mutant has not yet been considered. In this section we would like to provide a few basic insights for that case.

Quantitatively, one may guess that the chance of fixation in this regime behaves differently when *γ* < |*s*_0_| and *γ* > |*s*_0_|. In the latter case, the growth rate of a deleterious mutant is still positive (*γ* − |*s*_0_|) during half of the time, so the most probable (yet rare) route to fixation is based on picking a sequence of good years. During this series of lucky events the (on average) deleterious mutant plays the role of a beneficial one, and its time to fixation scales like ln *N*. Therefore, the chance of fixation, namely the chance to pick such a lucky sequence (which shrinks exponentially with the length of the sequence), decays like a power-law in *N*.

On the other hand, when *γ* < |*s*_0_|, the route to fixation involves an improbable series of successes in consecutive elementary duels (reflecting demographic stochasticity) and in such a case Π_*n*=1_ decays exponentially in *N*, like in the purely demographic case (1).

Looking at Eqs (13) and (24), one notices that the decay of Π_*n*=1_ when *s*_0_ < 0 (and *α* < 0) is due to the divergence of the ${G}^{-2\alpha }={G}^{2|{s}_{0}|/g}$ term in the denominator. Accordingly, our theory predicts in that regime (as suggested in^31^ for model A with $G\gg 1$) a power-law decay, ${{\rm{\Pi }}}_{n=1}\sim {N}^{-4|{s}_{0}|/{\gamma }^{2}\delta }$. The exponent of *N* grows with *s*_0_ and shrinks when the environmental stochasticity becomes stronger, as expected. However it does not show any qualitative shift when *γ* = |*s*_0_|.

This difficulty turned out to be related to the failure of the continuum approximation that has been used when we have translated the difference equation (4) to the differential equation (7). As explained in^32^, this procedure fails when the differences between neighboring points (say, Π_*n*+1_ − Π_*n*_) are too large and cannot be approximated using first and second derivatives only.

To overcome this obstacle a WKB approach was suggested^32^; here we would like to implement it for a model with environmental stochasticity. We shall neglect, for the moment, the effect of demographic noise and assume that extinction and fixation happen when the abundance reaches 1/*N* and 1 − 1/*N*, correspondingly.

As explained in the introduction [following Eq. (2)], in the absence of demographic noise and under model A dynamic $\dot{z}=sz$, where $z\equiv x/(1-x)$. Accordingly, during *δ* generations the dynamic of *z* satisfies,

27 $$z(t+\delta )=z(t){e}^{-(|{s}_{0}|\pm \gamma )\delta },$$

so one may consider the stochastic process as a biased random walk along the ln(*z*) axis. The random walker picks a left or a right move with equal chance 1/2, but left moves towards extinction ($\mathrm{ln}(z)\to \,\mathrm{ln}(z)-(|{s}_{0}|+\gamma )\delta \equiv \,\mathrm{ln}(z)-{\ell }_{1}$) are longer than right moves towards fixation ($\mathrm{ln}(z)\to \,\mathrm{ln}(z)+(\gamma -|{s}_{0}|)\delta \equiv \,\mathrm{ln}(z)+{\ell }_{2}$). The backward Kolmogorov equation is,

28 $${\rm{\Pi }}(\mathrm{ln}\,z)=\frac{1}{2}{\rm{\Pi }}(\mathrm{ln}\,z-{\ell }_{1})+\frac{1}{2}{\rm{\Pi }}(\mathrm{ln}\,z+{\ell }_{2}),$$

with the boundary conditions Π(ln *z* = −ln *N*) = 0 and Π(ln *z* = ln *N*) = 1.

To implement the WKB technique, one writes ${\rm{\Pi }}(\mathrm{ln}\,z)={e}^{f(\mathrm{ln}z)}$ and ${{\rm{\Pi }}}_{\mathrm{ln}z\pm \ell }={e}^{f(\mathrm{ln}z\pm \ell )}\approx {e}^{f(\mathrm{ln}z)\pm \ell f^{\prime} }$, where *f* ′ = ∂*f*(ln *z*)/∂ ln *z*. In this WKB formalism we implement the continuum approximation to *f*, i.e., for the logarithm of Π. Eq. (28) then takes the form,

29 $$1=\frac{1}{2}({e}^{-{\ell }_{1}f^{\prime} }+{e}^{{\ell }_{2}f^{\prime} }).$$

and yields the transcendental equation,

30 $$\cosh (\gamma \delta f^{\prime} )={e}^{|{s}_{0}|\delta f^{\prime} }.$$

Since *f* ′ is ln(*z*) independent, ${\rm{\Pi }}\sim \exp (f^{\prime} \,{\rm{l}}{\rm{n}}z)$, and given the boundary conditions one obtains,

31 $${\rm{\Pi }}({\rm{l}}{\rm{n}}\,z)=\frac{{e}^{f^{\prime} {\rm{l}}{\rm{n}}z}-{e}^{-f^{\prime} {\rm{l}}{\rm{n}}N}}{{e}^{f^{\prime} {\rm{l}}{\rm{n}}N}-{e}^{-f^{\prime} {\rm{l}}{\rm{n}}N}}.$$

If a “single mutant” is associated with *x*_0_ = 2/*N* (since we impose the boundary condition at 1/*N*, we have to define it that way, but the results must be independent of this choice) the chance of fixation decays like a power-law in *N*,

32 $${{\rm{\Pi }}}_{n=2}\sim {e}^{-2f^{\prime} \mathrm{ln}(N)}={N}^{-2f^{\prime} }.$$

**Figure 5:**
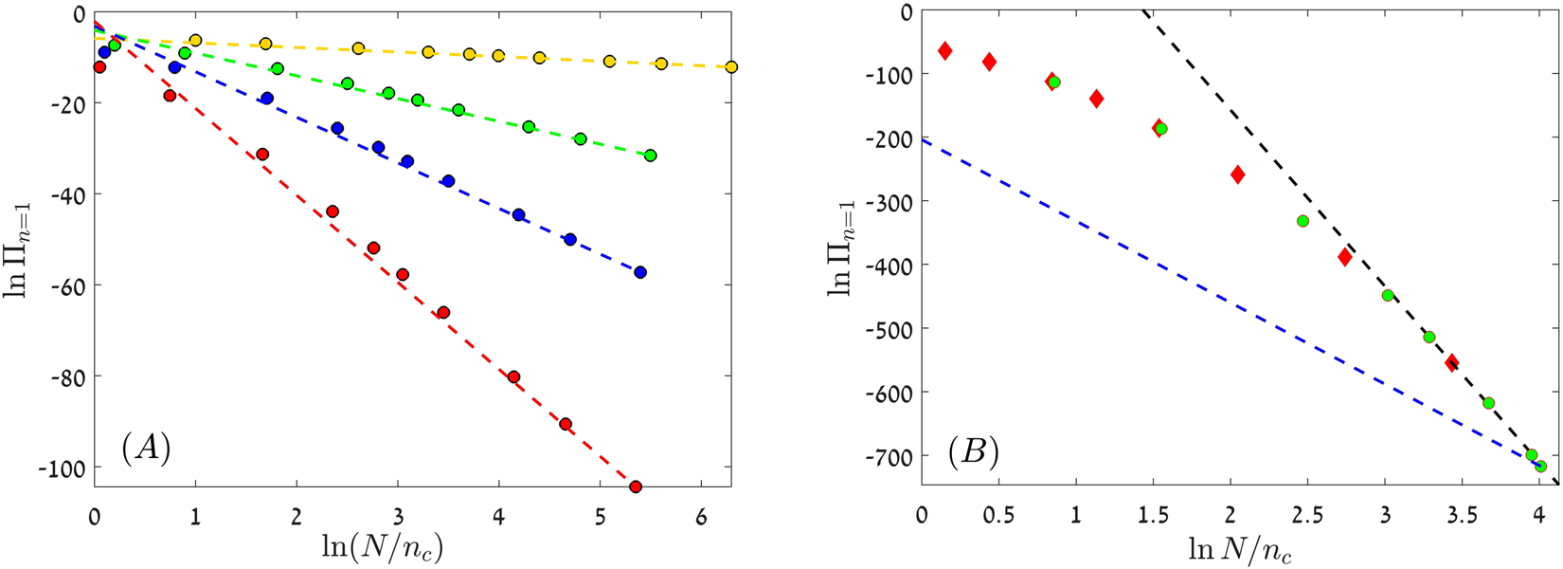
ln Π_*n*=1_ vs. ln *N*/*n*_*c*_ (*n*_*c*_ is defined with the absolute value of *s*_0_) for a deleterious mutant in the strong selection regime (${G}^{2|{s}_{0}|/g}\gg 1$). Panel (A) shows results for model A in the small *f* ′ regime. Parameters are *γ* = 0.2, *δ* = 0.1 and (−*s*_0_) takes the values 0.001 (yellow), 0.005 (green), 0.01 (blue) and 0.019 (red). Filled circles are the results obtained from the numerical solution of the discrete equation (4) and the dashed lines have the slope −4*s*_0_/*γ*^2^*δ*. Similar results were obtained for model B. In panel (B) the power of our WKB technique is demonstrated. Here *γ* = 0.25, *δ* = 0.1 and *s*_0_ = −0.2, model A results are presented as green circles while model B results are red diamonds. The slope suggested by the small *f* ′ approximation (blue dashed line, with slope −4*s*_0_/(*γ*^2^*δ*) = −128) clearly fails to describe the large *N* behavior. A much better fit is provided by the black dashed line, with a slope −2*f* ′ = −277 that was obtained from a numerical solution of Eq. (30). The intercepts of the dashed lines in both panels were chosen manually such that each line fits the last point of the corresponding dataset.

The value of *f* ′ is given by (30). For small *f* ′,

$$f^{\prime} \sim 2|{s}_{0}|/\delta {\gamma }^{2}=|{s}_{0}|/g,$$

in agreement with the definition of *C*_1_ above. On the other hand, if *f* ′ is large,

33 $$f^{\prime} \sim \frac{\mathrm{ln}\,2}{\delta (\gamma -|{s}_{0}|)},$$

and this expression diverges when *γ* → |*s*_0_|, as required, to mark the transition to the exponential phase. Between these two limits, the expression

$$f^{\prime} \sim \frac{2|{s}_{0}|}{\delta ({\gamma }^{2}-{s}_{0}^{2})},$$

provides a decent approximation. The accuracy of this WKB argument is demonstrated in Fig. 5.

This WKB argument gives the decay of Π in the power-law regime. When *γ* ≥ |*s*_0_| it fails, of course, since fixation of a deleterious mutant in this regime occurs only due to demographic stochasticity that was neglected in (28). For further discussion of the exponential phase (in the context of extinction times) see^33,34^.

## Discussion

Through this paper we dealt with the effect of environmental stochasticity on the fixation probability in a two-species zero-sum game. Two scenarios were discussed, one in which fitness fluctuations induce a stabilizing mechanism (model B) and one without this effect (model A). The results for both models were contrasted with the case of pure demographic noise (*γ* = 0) and with each other. Table 2 provides a summary of our main results.

Model A and model B have one important feature in common (Table 2, third line): the abundance scale *n*_*c*_ = *exp*[*g*/(2|*s*_0_|)]/*g*, below which the mutant population dynamic is dominated by fluctuations and above it by selection. This scale may become extremely large when the differences in the mean fitness are much smaller than the amplitude of the temporal fitness fluctuations, and one may easily imagine a situation where it becomes comparable or even larger than the effective size of an empirical community, meaning that the ecological or the evolutionary process takes place in the weak selection regime.

In the opposite, strong selection phase, the qualitative features of the chance of fixation Π_*n*=1_ are not much different between model A and model B. In both cases the chance of fixation for a beneficial mutant is *N*-independent (Table 2, sixth line) while the chance of fixation of a deleterious mutant falls like *N*^−2*f* ′^ when *γ* > |*s*_0_| (seventh line) and exponentially with *N* if *γ* < |*s*_0_|. In model A the chance of fixation in the strong selection regime decreases as the environmental noise becomes stronger, while in model B it decreases with the correlation time *δ* but increases with noise amplitude *γ*. (Through this discussion, when the features of model B are contrasted with those of model A, model B is assumed to support a noise induced attractive fixed point, i.e., $|\tilde{s}| < 1$. Otherwise, the behavior of model B dynamic is qualitatively the same as model A).

On the other hand, in the weak selection regime (Table 2, fifth line) there are substential differences between the two scenarios. As required by its name, in this regime selection is a second order effect and the fate of the mutant population is determined by stochasticity. In a stochastic and balanced game, like the classical gambler’s ruin problem, the chance to win is inversely proportional to the effective size of the community, so under purely demographic noise it is 1/*N* and under model A dynamic it is 1/ln *N*.

In sharp contrast with this result, in model B the system supports an attractive fixed point at x^*^. The plateau that characterizes Π(*x*) in that case (Fig. 3) reflects the effect of this attractive fixed point, marking the range of *x* values which lies in its basin of attraction. The attractiveness of this coexistence point grows with *N* and leads to an apparently paradoxical behavior: an increase of the chance of fixation with *N*. Once this fixed point becomes dominant, the fate of the mutant population depends on its chance of establishment Π_*est*_, i.e., of reaching the plateau, and on *C*_1_, the probability to jump from the plateau region to fixation. Since the plateau is wide, these two probabilities are *N* independent when the strength of selection *s*_0_ is relatively weak, and so is the chance of fixation itself. Even if *N* is huge, as long as it is much smaller than *n*_*c*_, model B yields an *N*-independent value for Π_*n*=1_ for both beneficial and deleterious mutants.

Even in the strong selection regime, where the chance of fixation of a deleterious mutant are vanishingly small, model B dynamic still supports an attractive fixed point at x^*^ as long as $|\tilde{s}| < 1$. The chance of establishment is still *N*-independent; it is the chance of fixation conditioned on establishment, *C*_1_, which goes to zero in that case. As shown in^18^, the lifetime of the mutant population, once established, is ${N}^{(1-|\tilde{s}|)/\delta }={N}^{1/\delta -\alpha }$ generations, a huge time for large communities. The stabilizing mechanism of model B thus allows for the long-term persistence of a macroscopic, ${\mathscr{O}}(N)$, extinction-prone population with negative fitness.

This phenomenon may provide a mechanistic explanation to one of the the mysteries of evolutionary dynamics: the ability of evolutionary pathways to cross fitness valleys, i.e., to sustain a chain of suboptimal intermediate forms that bridges between two fitness peaks in a rugged fitness landscape. This stochastic tunneling has been recognized a while ago as a major theoretical problem, since the chances for a tunneling event are vanishingly small^4^. To overcome this problem modern theoretical studies consider evolution on a neutral or nearly neutral (holey, high-dimensional, connected) fitness landscape. In this neutral picture a separate mechanism has to be invoked to explain speciation, while on rugged landscape each species corresponds to a separate fitness pick and disruptive selection is guaranteed by the landscape itself. The long term existence of macroscopic suboptimal populations, considered here in context of model B, may allow for such a tunneling to occur with relatively high probability through a chain of mutation as long as the depth of the fitness valley is smaller than *γ*^2^/2, while keeping the intermediate forms extinction-prone.

The relevance of the mechanisms suggested here to the development of natural communities depends on the amplitude of fitness variations with respect to their time-averaged value, on the typical correlation time of these fluctuations and on the range of competition - whether it is local/pairwise (model A) or global (model B). It is quite difficult to quantify *s*_0_ and *γ* from field data, and in experimental systems the external conditions are usually kept fixed, as opposed to the intrinsic variability of natural environments. Still, we believe that the theory presented here, when applied to some experiments and to field data in population genetics and community ecology, may suggest many new insights into the processes that govern the composition and the evolution of natural communities.
